# Effects of magnesium sulfate on the clinical course and GCS of patients with a severe diffuse axonal injury

**Published:** 2012-11

**Authors:** Moslem Shakeri, Firooz Salehpour, Ali Ahmadvand, Rozita Jafari, Abdolrasoul Safaiyan, Ali Meshkini

**Affiliations:** ^a^Tabriz University of Medical Sciences, Tabriz, Iran.; ^*b*^Imam Reza Teaching Hospital, Tabriz University of Medical Sciences, Tabriz, Iran.

## Abstract

**Background:**

Previous studies have shown that magnesium sulfate (MgSO4) administered in a patient with a diffuse axonal injury (DAI) can serve as a useful neuroprotective agent. The present study was conducted to examine whether magnesium sulfate has a therapeutic efficacy and safety in patients with a severe diffuse axonal injury.

**Methods:**

Adult patients admitted within 1 hour of a closed Traumatic Brain Injury (TBI) with a severe diffuse axonal injury that met eligibility criteria were randomly selected and divided into two groups. Our treatment guidelines consisted of an initial loading dose of 50 mg/kg magnesium sulfate and then 50 mg/kg QID (quarter in die) up to 24 hours after the trauma. The outcome measures were mortality, Glasgow Coma Scale (GCS) score, and motor function scores which were assessed up to 2 months post-trauma.

**Results:**

Magnesium showed a significant positive effect on GCS score at 2 months post-trauma (P=0.03). Motor functioning scores of patients in the MgSO4 group were higher than those in the control group but this was not statistically significant (P equal to 0.51).

**Conclusions:**

Findings of the present study demonstrated that administration of magnesium sulfate following severe DAI can have neuroprotective effect.

**Keywords:**

Severe diffuse axonal injury, Magnesium sulfate, Outcome


					Volume 4, Suppl. 1 Nov 2012
Publisher: Kermanshah University of Medical Sciences
URL: http://www.jivresearch.org
Copyright:
Congress of Iranian Neurosurgeons, 14-16 November 2012, Kermanshah, Iran
Paper No. 70
Effects of magnesium sulfate on the clinical course and GCS
of patients with a severe diffuse axonal injury
Moslem Shakeri a
, Firooz Salehpour a
, Ali Ahmadvand a
, Rozita Jafari b
, Abdolrasoul Safaiyan a
,
Ali Meshkini a,*
a
Tabriz University of Medical Sciences, Tabriz, Iran.
b
Imam Reza Teaching Hospital, Tabriz University of Medical Sciences, Tabriz, Iran.
Abstract:
Background: Previous studies have shown that magnesium sulfate (MgSO4) administered in a patient with a diffuse
axonal injury (DAI) can serve as a useful neuroprotective agent. The present study was conducted to examine
whether magnesium sulfate has a therapeutic efficacy and safety in patients with a severe diffuse axonal injury.
Methods: Adult patients admitted within 1 hour of a closed Traumatic Brain Injury (TBI) with a severe diffuse axonal
injury that met eligibility criteria were randomly selected and divided into two groups. Our treatment guidelines
consisted of an initial loading dose of 50 mg/kg magnesium sulfate and then 50 mg/kg QID (quarter in die) up to
24 hours after the trauma. The outcome measures were mortality, Glasgow Coma Scale (GCS) score, and motor
function scores which were assessed up to 2 months post-trauma.
Results: Magnesium showed a significant positive effect on GCS score at 2 months post-trauma (P=0.03). Motor
functioning scores of patients in the MgSO4 group were higher than those in the control group but this was not
statistically significant (P equal to 0.51).
Conclusions: Findings of the present study demonstrated that administration of magnesium sulfate following severe
DAI can have neuroprotective effect
Keywords:
Severe diffuse axonal injury, Magnesium sulfate, Outcome
* Corresponding Author at:
Ali Meshkini: Associate Professor of Neurosurgery, Tabriz University of Medical Sciences, Tabriz, Iran, Email: meshkinia@yahoo.com, (Meshkini A.).

				

